# The Effect of Self-Transcendence on Depression in Cognitively Intact Nursing Home Patients

**DOI:** 10.5402/2012/301325

**Published:** 2012-06-03

**Authors:** Gørill Haugan, Siw Tone Innstrand

**Affiliations:** ^1^Research Centre for Health Promotion and Resources HiST/NTNU, Faculty of Nursing, Sør-Trøndelag University College, 7004 Trondheim, Norway; ^2^Research Centre for Health Promotion and Resources HiST/NTNU, Department of Social Work and Health Science, Norwegian University of Science and Technology, 7491 Trondheim, Norway

## Abstract

*Aims*. This study's aim was to test the effects of self-transcendence on depression among cognitively intact nursing home patients. *Background*. Depression is considered the most frequent mental disorder among the elderly population. Specifically, the depression rate among nursing home patients is three to four times higher than that among community-dwelling elderly. Therefore, finding new and alternative ways to prevent and decrease depression is of great importance for nursing home patients' well-being. Self-transcendence is related to spiritual as well as nonspiritual factors, and it is described as a correlate and resource for well-being among vulnerable populations and at the end of life. *Methods*. A two-factor construct of the self-transcendence scale (interpersonal and intrapersonal) and the hospital anxiety and depression scale (HADS) was applied. A sample of 202 cognitively intact nursing home patients in central Norway was selected to respond to the questionnaires in 2008/2009. *Results*. A hypothesized SEM model demonstrated significant direct relationships and total effects of self-transcendence on depression. *Conclusion and Implication for Practice*. Facilitating patients' self-transcendence, both interpersonally and intrapersonally, might decrease depression among cognitively intact nursing home patients.

## 1. Introduction

In the next 30 years, the number of people over 65 in the world will almost double to 1.3 billion [1]. Moreover, the most rapidly growing segment is people over 80. By 2050 the percentage of those 80 and older will be 31%, while the number was 18% in 1980 [2]. This huge shift to an older population globally has given rise to the notions of the “third” and “fourth” ages in the lifespan development literature [3]. This differentiation of the last part of the lifespan into two separate phases is important because of the characteristic patterns of gains (growth) and losses (decline) seen in the “young old” and the “old old” [4]. For many of those in the fourth age (80+) issues such as physical illness and approaching mortality decimate their functioning and subsequently lead to the need for nursing home (NH) care. 

Depression is one of the most prevalent mentally ill health problems facing European citizens today [5], and it is considered one of the most frequent mental disorders in the elderly population. Moreover, depression is particularly common among these living in long-term care facilities [6]. Reported rates of major depression in older adults, depending on the location and characteristics of the sample, have ranged from 1% to 42% [7]. In general, the prevalence of late-life depression has been found to be higher in those patients with more medical illness. Specifically, rates of depression in NHs are three to four times higher than those in community-dwelling older adults [8]. Also, elderly who lack social/emotional support tends to be more depressed [9]. A review that included 36 studies from various countries reported a prevalence rate for major depression ranging from 6% to 26% and for minor depression from 11% to 50%. The prevalence rate of depressive symptoms ranged from 36% to 49% [10].

A linear increase of prevalence of depression with increasing age is described [11]. The three strongest explanatory factors on the age-effect of depression found are impairment, diagnosis, and somatic symptoms, respectively [11, 12]. Worse general medical health is seen as the strongest factor associated with depression among NH patients [13]. In general, NH patients are characterized by high age, frailty, mortality, disability, powerlessness, and dependency. Therefore, they are particularly vulnerable and more likely to become depressed. Moreover, studies in nursing homes report a large overlap of depression and anxiety [14–17]. The course of major depression and significant depressive symptoms in NH patients tends to be chronic, with a persistence rate of more than 50% of depressed patients still depressed after 6–12 months [18, 19].

Depression in NH patients is associated with negative outcomes such as impaired quality of life [20], impaired activities of daily living, substantial caregiver burden and worsened medical outcomes [21], increased risk of hospital admission [22], and a higher mortality rate [23]. A recent study reports that the presence of depression has a statistically and clinically significant negative impact on well-being [24]. The NH patients' sense of loss of independence and privacy, feelings of isolation and loneliness, the ever present death and grief, and lack of meaningful in-house activities are identified as risk factors for depression. Conversely, NH in-house activities and different kinds of mental health services have significant benefit for NH patients at risk for depression [25–27]. Accordingly, efforts to prevent and decrease depression are of great importance for NH patients' well-being and quality of life. The World Health Organization [28] has estimated that by 2020, depression is expected to be the highest ranking cause of disease in the developed world. Therefore, finding new and alternative approaches to weaken this detrimental development is highly warranted.

Self-transcendence is found to be such a correlate and a resource for well-being among vulnerable populations and at the end of life [29–31]. Self-transcendence includes a psycho-social-spiritual force toward personal maturity that is distinct from the more self-absorbed strivings for self-esteem and intimacy typical in earlier developmental phases. Hence, self-transcendence enhances an individual's searching for new perspectives, meaning, and well-being and allows him or her to overcome ego concerns. The idea of self-transcendence is inspired by human developmental theory emphasizing maturity as the developmental task in later life [32]. In accordance with this theory, the developmental crisis in old age entails integrity versus despair and is resolved by the development of inner resources as personal maturity and wisdom, qualities providing well-being. The developmental process of self-transcendence is stimulated by the challenges of age and coming to terms with death. Adults with higher levels of self-transcendence do not seek absolute answers to questions in life but seek meaning in life events as integrated within a moral, social, and historical context [33].

The central core of self-transcendence is described as connection between the individual, environment, and a transcendent being [33]. Self-transcendence refers to a multidimensional expansion of the self-boundaries intrapersonally (through self-acceptance and finding meaning in life), interpersonally (by reaching out to others or connecting with nature), transpersonally (by reaching out to a higher entity, being of purpose), and temporality (by integrating one's past and future into the present; a dynamic process involving adaption to physical, emotional, and/or spiritual distress). Within a holistic framework of the body, mind, and spirit as a whole, self-transcendence has been considered a central aspect of human spirituality [34]. As a general human dimension of personal maturity, self-transcendence has been related to spiritual and nonspiritual factors. Human spirituality is expressed and experienced in the context of caring connections with the self, others, nature, and a life force or God [35–39]. Accordingly, spirituality seems closely related to connectedness, the essence of self-transcendence. A recent study of the relationships between spiritual well-being and self-transcendence revealed that both interpersonal and intrapersonal self-transcendence significantly affect spiritual well-being in cognitively intact NH patients [40].

Self-transcendence has been studied in various disciplines, but it is of particular interest to nursing. The perspective of promoting health and well-being is fundamental and a major nursing concern in long-term care [41–43]. Studies on various populations demonstrate an inverse association between depression and self-transcendence [30, 31, 44–49]. Previous research demonstrates the significance of self-transcendence to older people well-being [31, 45, 50, 51]. Empirical support for the importance of self-transcendence for those at the end of life and facing their own mortality has been well documented [33, 47, 52–55].

In summary, the literature suggests depression as a common mental disorder among older people characterized by high age, impairment, and somatic symptoms. The patients' sense of loss of independence and privacy, feelings of isolation and loneliness, and lack of meaningful activities are risk factors for depression in NH patients. Self-transcendence is a resource for well-being among vulnerable individuals and at the end of life. A previous investigation of the dimensionality of the self-transcendence scale revealed that self-transcendence comprises one interpersonal and one intrapersonal factor [56]. Therefore, this study investigates the associations between interpersonal and intrapersonal self-transcendence and depression among cognitively intact NH patients. To the authors' knowledge, previous research has not examined the relationships between interpersonal and intrapersonal self-transcendence and depression in NHs by means of structural equation modelling (SEM). Our research question was *does self-transcendence affect depression in cognitively intact NH patients?* Based on the theoretical and empirical knowledge of depression and self-transcendence, the following hypotheses were formulated.

**Hypothesis 1**

Intrapersonal self-transcendence has negative effect on depression among cognitively intact NH patients.

**Hypothesis 2**

Interpersonal self-transcendence has negative effect on depression among cognitively intact NH patients.

## 2. Methods

### 2.1. Design and Ethical Considerations

The data were collected in 2008-2009 from 250 NH patients who met the inclusion criteria: (1) local authority's decision of long-term NH care, (2) residential time 6 months or longer, (3) informed consent competency recognized by responsible doctor and nurse, and (4) capable of being interviewed. The NH patients were approached by a head nurse they knew well. The nurse presented them with oral and written information about their rights as participants and their right to withdraw at any moment. Each participant provided informed consent. This population has difficulties completing a questionnaire on their own; therefore one-on-one interviews were conducted by three trained researchers and completed in the NH patients' private rooms. Researchers with identical professional background were chosen (RN, MA, trained and experienced in communication with elderly, as well as teaching gerontology at an advanced level) and trained to conduct the interviews as similarly as possible. Statistical correlational tests showed no significant differences between responses based on interviewers. The questionnaires relevant for the present study were part of a battery of nine questionnaires comprising 130 items. Interviewers held a large-print copy of questions and possible responses in front of the participants to avoid misunderstandings. Approval by the Norwegian Social Science Data Services was obtained for a license to maintain a register containing personal data (ref. no. 16443), and likewise we attained approval from The Regional Committee for Medical and Health Research Ethics in Central Norway (ref. no. 4.2007.645) as well as the directory of the 44 NHs.

### 2.2. Participants

The total sample consisted of 202 (80.8%) of 250 long-term NH patients representing 44 NHs. Long-term NH care was defined as 24-hour care; short-term care patients, rehabilitations patients, and cognitively impaired patients were not included. Participants' ages ranged from 65 to 104 years, with a mean of 86 years (SD = 7.65). The sample consisted of 146 women (72.3%) and 56 men (27.7%), where the mean age for women was 87.3 years and 82 years for the men. In total, 38 (19%) were married/cohabitant, 135 (67%) widows/widowers, 11 (5.5%) divorced, and 18 (19%) were single. The mean residential time when interviewed was 2.6 years for both sexes (range 0.5–13 years); 117 stayed in rural NHs, while 85 stayed in urban NHs. In all, 26.1% showed mild to moderate depression, only one woman scored 15+ indicating severe depression, 70.4% was not depressed, and nearly 88% had no anxiety disorder. Missing data was low in frequency: depression 5.0% and self-transcendence scale 5.9% and were handled by means of the listwise procedure.

### 2.3. Measures

*Depression* was assessed by the depression subscale of the hospital anxiety and depression scale (HADS), comprising seven items. Each item is scored from 0–3, and the maximum score is 21 on the subscale. The ranges of scores for cases are 0–7 normal, 8–10 mild disorder, 11–14 moderate disorder, and 15–21 severe disorder [57]. HADS has been tested extensively and has well-established psychometric properties [58]. In order to increase acceptability and avoid individuals feeling as though they are being tested for mental disorders, symptoms of severe psychopathology have been excluded. This makes HADS more sensitive to milder psychopathology [11]. HADS is translated into Norwegian and found to be valid for older people [11, 12]. Since the present study focuses on the associations between self-transcendence and depression, only the depression-items (subscale HADS-D) were included in the SEM model to be tested here. Sample items are “I still enjoy the things I used to enjoy,” “I can laugh and see the funny side of things,” “I feel cheerful,” “I feel as I'm slowed down,” “I have lost interest in my appearance,” and “I look forward with enjoyment to things.” The item “I can enjoy a good book or television-program” loaded very low (*λ* = 0.20, *R* ^2^ = 0.05), indicating less relevance for depression in this NH population and was dismissed. As the in-house activities in NHs are scarce, TV programs might be the only activity available, while reading books is difficult due to impairments. The items were scored on a four-point scale ranging from *totally disagree* to *totally agree*. The internal consistence of the construct (Table 1) was satisfactory (*α* = .68).

*Interpersonal *and* intrapersonal self-transcendence* were assessed by items from the self-transcendence scale (STS). The STS was developed from the 36-item Developmental Resources of Later Adulthood Scale [33, 59], which intended to identify intrapersonal, interpersonal, transpersonal, and temporal experiences characteristic of later life, reflecting expanded boundaries of self [46]. The STS comprises 15 items measuring characteristics of a matured view of life representing the extent to which a person expands personal boundaries. Each item is rated on a four-point Likert-type scale from 1.0 (*not at all*) to 4.0 (*very much*), with higher scores indicating higher ST (see the Supplemental Information in Supplementary Material available online at doi:10.5402/2012/301325). In previous studies, Cronbach's *α* ranged from.80 to.88 [31, 43, 48]. Content validity is adequate, based on a thorough review of empirical and theoretical literature [33]. Support for construct validity has been found in the relationships of self-transcendence scores to other measures [29, 60, 61]. 

The STS was translated into Swedish and back into English, and the back-translated version was then approved by the instrument constructor [62]. The Swedish version demonstrated internal consistency of.70–.85 (op.cit.) and was translated into Norwegian for the purposes of the present study. The Swedish and Norwegian language and culture are nearly identical in all aspects that matter this study. The STS is practically unchanged in the Norwegian version, but the words are spelled in Norwegian and the meanings have been checked. The two-factor construct of self-transcendence [56] was used, but the number of items was reduced; the item “letting go of my past losses” (ST15), which is reversed scored, loaded extremely low (*λ* = 0.11) and demonstrated *R* ^2^ = 0.02. This item was uncorrelated; therefore, there might be some translation problems and this item was dismissed. The items ”Having hobbies and interests I can enjoy,” “Being involved with other people,” “Sharing my wisdom or experience with others,” “Helping others in some way,” “Having an ongoing interest in learning,” “Able to move beyond things that once seemed so important,” and “Finding meaning in my spiritual beliefs” were indicators for interpersonal self-transcendence, while the items “Accepting myself as growing older,” “Adjusting well to my present life situation,” “Adjusting well to changes in my physical abilities,” “Finding meaning in my past experiences,” “Accepting death as a part of life,” “Letting others help me when I may need it,” and “Enjoying my pace of life” constituted the intrapersonal ST construct. Cronbach's *α* in current study was 0.72 (all 15 items), while *α* for interpersonal self-transcendence (seven items) was .76 and .65 for intrapersonal self-transcendence (seven items) (Table 1). Reliability for the latent constructs in this study is further investigated inside the confirmatory factor analysis (CFA). Composite reliability (*ρ*_*c*_) is reported in Table 2 displaying values between 0.65 and 0.75; values greater than 0.60 are desirable [63].

### 2.4. Statistical Analysis

A structural equation model (SEM) of the hypothesized relations between the latent constructs of depression and self-transcendence was tested by means of LISREL 8.8 [64]. Using SEM accounts for random measurement error, and the psychometric properties of the scales in the model are more accurately derived. Since the standard errors are estimated under nonnormality, the Satorra-Bentler-scaled chi-square statistic was applied as a goodness-of-fit statistic, which is the correct asymptotic mean even under nonnormality [65]. In line with the rules of thumb of conventional cut-off criteria [66], the following fit indices were used to evaluate model fit: chi-square (*χ* ^2^)—a small *χ* ^2^ and a nonsignificant *P* value corresponds to good fit [64]. Also, we used the root mean square error of approximation (RMSEA) and the standardized root mean square residual (SRMS) with values below 0.05 indicating good fit, while values smaller than 0.08 are interpreted as acceptable [66, 67]. The comparative fit index (CFI) and the nonnormed fit index (NNFI) with an acceptable fit at 0.95 and good fit at 0.97 and above were used, and the normed fit index (NFI) and the goodness-of-fit index (GFI) with an acceptable fit at 0.90, while a good fit was set to 0.95. For the adjusted GFI (AGFI), acceptable fit was set to 0.85 and good fit at 0.90 (op.cit.).

Before examining the hypothesized relationships in the SEM analysis, the measurement models were tested by confirmatory factor analysis (CFA). The CFA provided a good fit to the observed data for the interpersonal self-transcendence (*χ* ^2^ = 22.28, *P* < 0.073, RMSEA = 0.055, SRMR = 0.047, NFI = 0.95, NNFI/CFI = 0.97/0.98, GFI/AGFI = 0.96/0.92), intrapersonal self-transcendence (*χ* ^2^ = 15.77, *P* < 0.0033, RMSEA = 0.026, SRMR = 0.056, NFI = 0.93, NNFI/CFI = 0.99/0.99, GFI/AGFI = 0.96/0.92), and the depression scale (*χ* ^2^ = 9.43, *P* < 0.46, RMSEA = 0.016, SRMR = 0.039, NFI = 0.96, NNFI/CFI = 1.00/1.00, GFI/AGFI = 0.98/0.95). All parameter estimates were significant (*P* < 0.05) and loaded positively and clearly on their intended latent variable with standardized factor loadings between 0.22 and 0.77. For scaling the variances of the dependent latent variable was set at 1.

## 3. Results

### 3.1. Descriptive Analysis

Table 1 displays the means (M), standard deviations (SD), Cronbach's alpha, and correlations matrix for the constructs of self-transcendence (interpersonal and intrapersonal) and depression. The correlations between the measures were in the expected direction. Moderate correlations were found between the latent constructs included in the SEM model (Table 1). The alpha levels for the various measures indicate an acceptable level of inter-item consistency in the measures [68] with Cronbach's alpha coefficients of .65 or higher.

### 3.2. Structural Equation Modeling (SEM)

**Figure 1:**
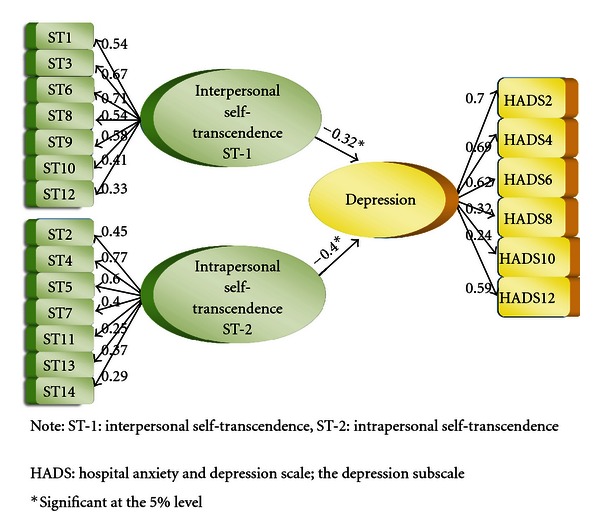
SEM Model 1. Measurement models and directional relationships for self-transcendence (ST) to depression.

In order to investigate how interpersonal self-transcendence (ST-1) and intrapersonal self-transcendence (ST-2) relate to depression, Model 1 comprising six depression, seven interpersonal self-transcendence and seven intrapersonal self-transcendence items was estimated.  Figure 1 shows Model 1 with its measurement and structural models. All estimates were significant (*P* < 0.05), and the factor loadings ranged 0.22–0.72 and *R* ^2^-values 0.05–0.59. Model 1 fitted well (*χ* ^2^ = 201.64, *P* = 0.035, df = 167, RMSEA = 0.034, *P* value = 0.96, NNFI = 0.96, CFI = 0.97, SRMR = 0.067, and AGFI = 0.85). However, NFI = 0.84 and GFI = 0.88 were low. Traditionally, a cut-off point of 0.90 has been recommended for the GFI and NFI. However, the AGFI, which adjusts the GFI by degrees of freedom, is acceptable. Also the NFI is sensitive to sample size, underestimating fit for samples less than 200 [69]; therefore researchers should not rely solely on the GFI and NFI [70].

Table 3 shows the standardized regression coefficients of the directional relationships and the total effects between the latent constructs in Model 1. As hypothesized, both directional paths were significant displaying an inverse relationship, from interpersonal self-transcendence to depression (*γ*1,1 = −0.30*) and from intrapersonal self-transcendence to depression (*γ*1,2 = −0.41*) (Table 3).

A scrutiny of the total effects of interpersonal self-transcendence and intrapersonal self-transcendence on depression revealed statistical significant total effects from both interpersonal and intrapersonal self-transcendence on all the depression items (Table 3). No indirect effects were displayed.

## 4. Discussion

The World Health Organization [28] estimated that, by 2020, depression will be the highest ranking cause of disease in the developed world. Therefore, finding new and alternative approaches to weaken this development is necessary. Moreover, a huge shift to an older population worldwide is highlighted, and the most rapidly growing segment is that of people over 80 years old. Simultaneously, depression is one of the most frequent mental disorders in the elderly population and specifically among NH patients. Therefore, finding new interventions to increase well-being and decrease depression among NH patients is highly warranted. The aim of this study was to explore the association between interpersonal and intrapersonal self-transcendence and depression in cognitively intact nursing home patients. By doing so we sought to contribute to a holistic nursing perspective of promoting well-being in NH patients in two ways: first, this study supplies empirical knowledge to the growing body of self-transcendence knowledge by exploring self-transcendence among NH patients; second, by applying a two-factor construct of self-transcendence [56] that has been shown to be psychometrically superior the one-factor construct, the present study allows a more complex examination of the associations between self-transcendence and depression. The present study examines the relationships and the influences of interpersonal and intrapersonal self-transcendence on depression in NH patients. By means of advanced statistical analysis such as structural equation modeling (SEM), this study provides more specific guidelines to nursing interventions promoting well-being in NH patients and suggests that finding ways to enhance an individual's intrapersonal and interpersonal self-transcendence might be beneficial in this matter. 

More specifically, we found that the hypothesized relationship between self-transcendence (intrapersonal and interpersonal) and depression was fully supported. As hypothesized, interpersonal self-transcendence was negatively associated with depression. It is reasonable that interpersonal self-transcendence including personal interests, learning, involvement with others, connectedness, sharing one's wisdom, and helping others is associated with low depression. Therefore, nursing interventions facilitating NH patients' involvement and connectedness, sharing wisdom, and possibilities helping others might decrease and prevent depression.

Also, the intrapersonal self-transcendence comprising NH patients' self-acceptance as growing older, adjusting well to the changes in physical abilities and the present situation, and finding meaning in one's past experiences related negatively to depression. The statistical tests suggested that the intrapersonal self-transcendence affects depression more than the interpersonal self-transcendence among cognitively intact NH patients. Therefore, staff nurses' awareness of NH patients' self-acceptance and adjustment to the life situation is essential for well-being as well as enjoyment, humor, and laughter. Supporting NH patients' effort to accept themselves and adjust well to the life situation and one's declines is significant to decrease and prevent depression. 

At 86 years old (mean age in the present study), representing the “old old” [4] living in a NH represents a life perspective where openness to keep learning, enjoying hobbies and interests, and involving with others might seem less important than accepting oneself and adjusting well to the “here-and-now” in the NH environment. Loss of functions, relationships, privacy, self-determination, and connectedness and the forthcoming death, together with searching for meaning in the in-house institutionalized NH life, seem in general as the main challenges. Generally NH patients are in great need of care and assistance because of physical declines and other limitations, and they represent a vulnerable group. From this particular point of view, NH patients' energy to create good experiences seems low. Therefore, these individuals need initiative and vitalization from others and the environment. Elderly who lack social/emotional support tend to report more depression [9]. Consequently, the nurse-patient relationships might be an overriding figure in the NH patients' daily life and might serve as a vital resource of human involvement, interaction, connectedness, and a facilitator of meaningful in-house activities for NH patients [71].

Few social relations for dialogue, self-reflection, and connectedness are left; in our sample only 19% had a partner. The social climate in a NH environment is largely determined by the staff-patient interactions that take place within it [72]. The potentials for self-transcendence and well-being are important considerations in NH care; the participants had stayed in NH for 6 months or longer. Because the length of stay is long, much time is available to enter into meaningful relationships and communication with patients, pursuing appropriate interventions to promote self-transcendence. Within Erikson's model of human psychosocial development [32], adulthood is concerned with generativity versus stagnation and maturity with ego integrity versus despair. Therefore, self-transcendence as an individual developmental process toward personal maturity and a resource for well-being might serve as an indicator of successful aging among NH patients.

Some of the intrapersonal self-transcendence indicators loaded low and displayed low *R* ^2^-values. These items involve finding meaning in past experiences, accepting death as a part of life, letting others help, and enjoying one's pace of life. Being in the “fourth age,” living in a NH, represents a life situation where letting others help and accepting death are quite obvious. NH patients' daily life is strongly influenced by the routines and availability of help and support from the staff nurses. In this regard, NH patients might find that they do not have their own pace of life, and enjoying one's pace of life seems less relevant. Also the items “I feel as I'm slowed down” and “I have lost interest in my appearance” loaded low, but the composite reliability was good. The low values might indicate that these items are less relevant for this particular sample.

## 5. Limitations

This study expands previous studies by testing the associations between the two-factor construct of self-transcendence and depression among NH patients by using structural equation modeling. Using SEM accounts for random measurement error and the psychometric properties of the scales in the model are more accurately derived. Nevertheless, the findings of this study must be discussed with some limitations in mind.

First, Model 1 comprises 20 variables, indicating a desirable *N* = 200, while in the present study *N* = 185. A larger sample would significantly increase the statistical power of the tests. A second limitation concerns the use of self-reported data, which implies a certain risk that the findings are based on common-method variance [73].

Moreover, the fact that the researchers visited the participants to help fill in the questionnaires might have introduced some bias into the respondents' reporting. The questionnaires were part of a battery of nine questionnaires with 130 items. Therefore, frail older NH patients might become exhausted when completing the questionnaires, which may cause a possible bias. To avoid such a bias, experienced researchers were carefully picked and trained in conducting the interviews following a standardised procedure, including small breaks on specific points during the interview process. This procedure worked out very well; in just three cases the interviews had to be completed the next day due to respondent's fatigue. Actually, most participants were even more vigorous after completing the interview.

The NFI and GFI were lower than the recommended value of 0.90 for both indices, indicating uncertainty regarding the associations in Model 1. However, the NFI and GFI are sensitive to small samples, underestimating fit for samples less than 200; therefore they should not be relied on solely [70]. Nevertheless, the CFI is a revised form of the NFI that considers the sample size and performs well even when sample size is small [74]. CFI in the present study was 0.96, supporting Model 1. In addition, AGFI is an adjusted form of GFI that accounts for the degrees of freedom and was inside the recommended cut-off value. 

## 6. Relevance to Clinical Nursing

According to the European Commission's Green Paper on mental health [5], depression is one of the most prevalent mental health problems facing European citizens today. Taking into account the highly chronic nature of these psychological states, we consider our findings noteworthy in their suggestion that self-transcendence might impair the level of depression. Knowledge of how self-transcendence and depression relate to each other in this respect is important for researchers, nurses, and clinicians.

This study demonstrates that both intrapersonal and interpersonal self-transcendence influence depression. Accordingly, facilitating nursing intervention to provide patients' self-transcendence would promote integrity and well-being and prevent despair and depression. The results suggest that the intrapersonal self-transcendence affects depression stronger than the interpersonal self-transcendence among cognitively intact NH patients. This might indicate that facilitating self-acceptance and helping patients adjust well are crucial to decreasing depression among NH patients. Furthermore, connecting with others, sharing wisdom, and helping others when possible appear as vital to preventing depression and increasing well-being in cognitively intact NH patients.

Due to a combination of factors such as patients' communication impairment, clinicians' focus on treating medical conditions, normalization of depression in later life, and a lack of training in mental health among staff in NHs, depression can easily go undetected among the NH population [75, 76]. Therefore, self-transcendence might serve as a framework for staff nurses' awareness, assessing patients' mood, and connectedness resources. Staff nurses are increasingly aware that good nursing care consists of more than the competent performance of a number of nursing activities. However, for many nurses, it is much less clear what this “more” means and what importance it has in nursing. Nurses may encourage self-transcendence by facilitating patients' connections with others, stimulating inner reflections and connection to their inner thoughts and emotions, and by promoting spiritual faith, facilitating hobbies, helping others, and sharing wisdom. Enhancing acceptance of the self, death, and one's life situation might prevent and decrease depression among NH patients. As connectedness is described as the core of self-transcendence, offering connectedness might be a central aspect of NH care. The interpersonal relationship in patient-nurse interactions has been found to be an essential factor of quality of care, as perceived by long-term care patients [71, 77, 78]. Nurse-patient interaction can help NH patients preserve their dignity, identity, and integrity [79]. By means of listening to the patients, communicating, and treating the patients with respect, by using empathic understanding, and acknowledging him/her as a person who is to be taken seriously and attended to, staff nurses might promote self-transcendence and therefore personal maturity, decreased depression, and enhanced well-being [71, 80–84].

Nursing research and education should pay more attention to interventions promoting self-transcendence and well-being in order to develop a more comprehensive and practice-based view of good nursing care that inspires NH staff nurses as they perform their daily care practices. More research of the effectiveness of such strategies is highly needed. Educational nursing curricula should underline and facilitate nurse-patient interaction and interventions to expand NH patients' personal self-boundaries. Advancing the staff nurses' presence with the patient might contribute to increased self-transcendence and decreased depression.

## 7. Conclusion

The present study suggests that self-transcendence negatively and significantly affects depression in cognitively intact NH patients. Depression was significantly influenced by the interpersonal and the intrapersonal self-transcendence. Consequently, nursing interventions and in-house activities aiming to increase patients' self-transcendence might have a great impact on NH patients' depression and their well-being.

## Supplementary Material

The two appendixes show the measurement instruments for the Hospital Anxiety and Depression Scale (HADS) and self-transcendence with *means* and *standard deviation* (*SD*).

## Figures and Tables

**Table 1:** Means (M), standard deviations (SD), Cronbach's alpha, and correlation coefficients for the study variables.

Construct	M	SD	Cronbach's alpha	ST-1	ST-2	HADS-D
ST-1 (7 items)	2.54	0.58	.76	—		
ST-2 (7 items)	3.04	0.42	.65	0.29*	—	
HADS-D (6 items)	2.32	0.39	.68	0.43*	0.50*	—
Self-transcendence (15 items)	2.85	0.34	.72			

Note: ST-1: interpersonal self-transcendence. ST-2: intrapersonal self-transcendence. HADS-D: depression. *Significant at the 0.05 level.

**Table 2:** Measurement models included in Model 1: interpersonal self-transcendence (ST-1) and intrapersonal self-transcendence (ST-2) to depression (HADS-D). Standardized factor loadings and *t*-values. Squared multiple correlations (*R* ^2^). Composite reliability^1^ (*ρ*_*c*_).

Items	Parameter	LISREL estimate	*t*-value	*R* ^2^
ST-1				
ST1	*λx*1,1	0.54	5.95*	0.28
ST3	*λx*3,1	0.67	9.39*	0.44
ST6	*λx*6,1	0.71	9.02*	0.52
ST8	*λx*8,1	0.54	6.12*	0.30
ST9	*λx*9,1	0.58	7.85*	0.33
ST10	*λx*10,1	0.41	4.65*	0.16
ST12	*λx*12,1	0.33	3.90*	0.11

ST-2				
ST2	*λx*2,2	0.45	4.66*	0.20
ST4	*λx*4,2	0.77	7.55*	0.59
ST5	*λx*5,2	0.60	6.08*	0.37
ST7	*λx*7,2	0.40	3.88*	0.16
ST11	*λx*11,2	0.25	2.58*	0.06
ST13	*λx*13,2	0.37	3.60*	0.14
ST14	*λx*14,2	0.29	2.53*	0.08

HADS-D				
HADS2	*λy*1,1	0.70	—	0.49
HADS4	*λy*2,1	0.70	7.48*	0.49
HADS6	*λy*3,1	0.62	5.36*	0.39
HADS8	*λy*4,1	0.32	3.88*	0.10
HADS10	*λy*5,1	0.22	2.47*	0.05
HADS12	*λy*6,1	0.58	5.27*	0.34

*ρ*_c_ ST-1 7 items	*ρ*_c_	0.75	—	—
*ρ*_c_ ST-2 7 items	*ρ*_c_	0.65	—	—
*ρ*_c_ HADS-D 6 items	*ρ*_c_	0.71	—	—

Note. *P < 0.05; ^1^composite reliability *ρ*_*c*_ = (∑*λ*)^2^/[(∑*λ*)^2^ + ∑(*θ*)].

**Table 3:** SEM analysis. Model 1^1^. Standardized gamma^2^. Total^3^ effects of self-transcendence (ST) on nursing home patients' depression.

Construct	Parameter		LISREL estimate	*t*-value
ST-1 to HADS-D	*γ* 1,1		−0.32	−3.01*
ST-2 to HADS-D	*γ* 1,2		−0.40	−3.43*
Total effects of self-transcendence on depression (HADS−D)				
	ST-1	*t*-value	ST-2	*t*-value
HADS2	−0.18	−3.01*	−0.23	−3.43*
HADS4	−0.18	−3.13*	−0.23	−3.30*
HADS6	−0.15	−2.93*	−0.19	−3.07*
HADS8	−0.11	−2.87*	−0.14	−2.64*
HADS10	−0.08	−1.88	−0.10	−2.41*
HADS12	−0.17	−2.40*	−0.22	−4.33*

Note. *Significant at the 5% level. ^1^Model 1: comprising six HADS variables and 10 ST variables. ^2^Gamma standardized regression coefficients representing directional relationships between ST and depression. ^3^Total effect represent the total influence of the explanatory variable on depression.
