# Supplementary material for: The Effect of Self-Transcendence on Depression in Cognitively Intact Nursing Home Patients
Source: ISRN Psychiatry. 2012 Jun 3;2012:301325. doi: 10.5402/2012/301325 (PMC3658806; doi:10.5402/2012/301325)
Supplement: Supplementary file 1 — The two appendixes show the measurement instruments for the Hospital Anxiety and Depression Scale (HADS) and self-transcendence with means and standard deviation (SD). [file 301325.f1.doc]

**Supplemental Information**

**Appendix**

| **Appendix 1.** Measurement instrument; Hospital Anxiety and Depression Scale (HADS) Depression Subscale. Means score and standard deviation. | | |
| --- | --- | --- |
| **HADS item** | *Mean* | *SD* |
| HADS2 I still enjoy the things I used to enjoy | .879 | .822 |
| HADS4 I can laugh and see the funny side of things | .621 | .845 |
| HADS6 I feel cheerful | .533 | .759 |
| HADS8 I feel as I’m slowed down | 1.209 | 1.075 |
| HADS10 I have lost interest in my appearance | .803 | 1.079 |
| HADS12 I look forward with enjoyment to things | .864 | .943 |
| HADS14 I can enjoy a good book or TV program | .561 | .857 |
| HADS Depression Sum Score | 5.57 | 3.238 |
| *Note:* The items were scored on a four-point scale ranging from totally disagrees to totally agree. | | |

| **Appendix 2**. Self-Transcendence (ST): *Means* and standard deviation (*S.D*.) | | |
| --- | --- | --- |
| **ST Items** | ***Mean*** | ***S.D.*** |
| ST1. Having hobbies and interests I can enjoy. | 2.41 | .899 |
| ST2. Accepting myself as I grow older. | 3.09 | .548 |
| ST3. Being involved with other people or my community when possible. | 2.63 | .878 |
| ST4. Adjusting well to my present life situation. | 3.10 | .595 |
| ST5. Adjusting to changes in my physical abilities. | 2.90 | .621 |
| ST6. Sharing my wisdom or experience with others. | 2.45 | .795 |
| ST7. Finding meaning in my past experience. | 3.06 | .660 |
| ST8. Helping others in some way. | 2.64 | .816 |
| ST9. Having an ongoing interest in learning. | 2.32 | .959 |
| ST10. Able to move beyond some things that once seemed so important. | 2.78 | .788 |
| ST11. Accepting death as a part of life. | 3.11 | .599 |
| ST12. Finding meaning in my spiritual beliefs. | 2.53 | 1.00 |
| ST13. Letting others help me when I may need it. | 3.22 | .511 |
| ST14. Enjoying my pace of life. | 2.88 | .617 |
| ST15. Letting go of my past losses. | 3.38 | .802 |
| ST total mean score | 2.83 | .352 |
| *Note*: The STS is based on a four-point scale ranging from 1 (not at all), 2 (very little), 3 (somewhat) to 4 (very much). | | |
